# The psychometric properties of the respectful maternity care (RMC) for an Iranian population

**DOI:** 10.1186/s12913-020-05729-x

**Published:** 2020-09-22

**Authors:** Khadije Hajizadeh, Mohammad Asghari Jafarabadi, Maryam Vaezi, Shahla Meedya, Sakineh Mohammad-Alizadeh-Charandabi, Mojgan Mirghafourvand

**Affiliations:** 1 Students’ Research Committee, Midwifery Department, Tabriz University of Medical sciences, Tabriz, Iran; 2 Road Traffic Injury Research Center, Faculty of Health, Tabriz University of Medical Sciences, Tabriz, Iran; 3 Fellowship of gynecology oncology, Alzahra teaching hospital, Tabriz University of Medical Sciences, Tabriz, Iran; 4 Member of South Asia Infant Feeding Research Network (SAIFRN), School of Nursing, Faculty of Science, Medicine and Health, University of Wollongong, Wollongong, Australia; 5 Department of Midwifery, Faculty of Nursing and Midwifery, Tabriz University of Medical Sciences, Tabriz, Iran; 6 Social Determinants of Health Research Center, Tabriz University of Medical Sciences, Tabriz, Iran

**Keywords:** Respectful, Maternity care, Validity, Reliability, Psychometric, Iran

## Abstract

**Background:**

The absence of Respectful Maternity Care (RMC) deters mothers from seeking maternity care services. Given the importance of RMC and the lack of a standard tool for its assessment in Iran, the present study was conducted to translate and assess the psychometric properties of the RMC questionnaire in Iranian women.

**Methods:**

Forward-backward method was used for translating the questionnaire from English into Persian. A total of 265 postpartum women entered the study by simple random sampling from public and private hospitals in Tabriz, Iran. The validity of the questionnaire was confirmed through the face, content and construct validity. Construct validity was assessed through exploratory and confirmatory factor analyses. The internal consistency and test-retest reliability were used to confirm the reliability of the questionnaire. Internal consistency was examined by measuring the Cronbach’s alpha in a sample of 20 mothers, and test-retest stability by calculating the Intraclass Correlation Coefficient (ICC) in the same group of mothers, who had completed the questionnaire twice with a two-week interval.

**Results:**

The exploratory factor analysis led to the extraction of one factor. Item 12 was eliminated due to its low factor loading. X^2^/df was less than 5, and RMSEA was less than 0.08, which confirms the validity of this model. The Cronbach’s alpha coefficient was obtained as 0.93 and ICC (with 95% confidence interval) as 0.98 (0.96 to 0.99).

**Conclusion:**

The results of the study demonstrated that the Iranian RMC scale can be used as a valid and reliable instrument to assess RMC in Iran.

## Background

Maternal and neonatal health is one of the high priorities for the World Health Organization to reduce maternal mortality and morbidity. Having a skilled birth attendant (SBA) is part of maternal mortality prevention programs [1]. According to the UNICEF reports, only 68% of all women worldwide have an SBA during childbirth. In developed countries, nearly all women have access to SBAs before and during childbirth and in the postpartum period, while only 55% of women have access to SBAs in developing countries [2, 3]. According to sustainable development goal three, a key strategy for reducing the high rate of maternal and neonatal morbidity and mortality is to increase deliveries by SBAs [4]. However, mistreatment of patients by healthcare personnel is an issue has been reported in both high- and low-income countries [5]. Women’s satisfaction with maternity care is closely linked to the healthcare personnel’s behaviors [4].

The mistreatment of women by healthcare personnel can influence women’s satisfaction with birth and the care that she receives [4]. These mistreatment behaviors include physical and verbal abuse, non-consented care, neglecting the mother, taking bribes, discrimination, disrespectful care and non-confidential care [5]. Such mistreatments are a serious violation of human rights, since women are physiologically, socially and psychologically more vulnerable during labor and childbirth [6–8].

Evidence suggests that Disrespect and Abuse (D&A) in maternity care may deter mothers from seeking maternity care [9]. Many health experts and stakeholders believe that D&A is a major impediment in maternal facilities. However, evidence demonstrated that Respectful Maternity Care (RMC) can reduce maternal mortality and morbidity [9, 10]. Therefore, assessment of RMC by valid questionnaires is necessary for promoting of maternal health [11].

The RMC tool was first developed by Sheferaw et al. in 2016 [11] in two phases, including qualitative (in-depth interviews) and quantitative (expert assessments through interviews and emails by trained people) phases, on 509 women immediately after childbirth until 7 weeks later in 11 urban health facilities. Following the review of literature and in-depth interviews with women, seven dimensions with five to 12 items each (making for a total of 60 items) were extracted. After eliminating a number of the items, 15 items were approved in four dimensions, namely abuse-free care, friendly care, discrimination-free care and timely care [11]. Considering the importance of RMC and the fact that D&A can violate human rights and affect women’s choice of delivery type and exacerbate the mother’s psychological problems, and also there were no evidence of RMC quality measurement in Iran before starting this study, the present study was conducted to translate and assess the psychometric properties of RMC for use in Iranian women.

## Methods

### Aim

The present study aims to adapt RMC to the Iranian culture and determine its psychometric properties.

### Study’s participants

The inclusion and exclusion criteria for this study have been published previously in another article [12].

### Sample size

Selecting of ten participants per item has been suggested for factor analysis by Nunnally and Bernstein [13]. Because the RMC scale has 15 items, therefore, 150 participants were needed. With cluster sampling and a design effect of 1.5, the sample size was found as 225, which was increased to 265 to take account of a potential withdrawal rate of 20%.

### Tool

The detailed information about the tool has been published in the protocol paper [14].

### Translation process

First, written permission for adapting the tool to the Iranian culture was obtained from the tool developer (Sheferaw). The original version of the tool was translated from English into Persian by a native English speaker who was also competent in Persian language. The translated version was reviewed by the research team, and then translated back from Persian into English. This step of the translation was carried out by two translators competent in both languages who had not been involved in the forward translation. Next, this translated version was reviewed by two people familiar with specialized concepts and competent in both languages and the final version was thus obtained [15]. The Persian and English versions are available as appendix 1 and 2, respectively.

### Data collection

The study was conducted in the postpartum ward of public (Alzahra, Taleghani) and private (Behbood, Nor-e-Nejat, and Shahriyar, 29 Bahman) hospitals in Tabriz. A total of 265 postpartum women were selected. The questionnaire included socio-demographic, obstetrics characteristics, and the RMC scale. The demographic questionnaire used contained questions on the mother’s age, education, occupation, income, the neonate’s gender and pregnancy type (intended or unintended). The validity of this questionnaire was confirmed using content validity.

### Face and content validity

To determine the face validity of the scale, 20 postpartum women were invited to assess all the items in terms of simplicity, clarity and relevance. Then, based on their responses and the Likert-type scale (from 1 point = ‘totally difficult or unclear’ to 4 points = ‘totally simple and clear’), the item impact was determined for each item using the following formula: Impact = Importance (mean responses to the item) × Frequency (the number of responses with the score of four). The items scoring less than 1.5 are eliminated [15].

Content validity was determined by both a quantitative and a qualitative method. In the qualitative method, ten experts in reproductive health, midwifery and psychiatric nursing were asked to assess the translation of each item in terms of grammar, use of appropriate terms and correct placement of the items and to present their corrective comments. In the quantitative method, the Content Validity Ratio (CVR) and Content Validity Index (CVI) were measured. To determine the CVI, the items were assessed in terms of relevance, clarity and simplicity using a 4 point Likert scale. Scores above 0.79 were considered acceptable. CVR was determined by experts who asked to evaluate each item in terms of importance using a 4 point Likert scale. The minimum CVR was taken as above 0.62 based on Lawshe’s table.

### Construct validity

Exploratory (EFA) and confirmatory (CFA) factor analyses were used to assess the construct validity. Bartlett’s test, the Kaiser-Meyer-Olkin (KMO) index, scree plots and Oblimin rotation were used in EFA. The adequacy of the data for conducting EFA is confirmed based on values above 0.7 [16]. The factors were extracted by Principal Component Analysis (PCA) and varimax rotation, and the number of factors was determined based on Eigen Values (EVs) and scree plots. EV determines what proportion of variance in the total data is explained by one factor. Therefore, higher EVs for any factor increase the proportion of variance explained by that factor [17].

Factor analysis assesses the intra-variable relationships and is used to extract categories of items most related to each other. The items with a factor loading lower than 0.3 were considered as candidates for elimination, and then the research team decided about whether or not to keep the items where they were greater than 0.3 and less than 0.5. Also, the factors’ consistency with the subscales of the original scale was assessed after the extraction of each factor and the items in the factor.

The structure of the extracted factors was assessed using the EFA model and CFA. The indices were used for assessing the exploratory model’s fit. Fit indices and reasonable values of theses indices for CFA were considered as Comparative Fit Index (CFI) ≥0.90, Root Mean Square Error of Approximation (RMSEA) < 0.08, X^2^/df < 5, Tucker-Lewis Index (TLI) ≥0.95 and also, Comparative Fit Index (CFI), Goodness of Fit Index (GFI), Adjusted Goodness of Fit Index (AGFI), Normed Fit Index (NFI) and Incremental Fit Index (IFI) > 0.9 [18].

Internal consistency and test-retest stability methods were used to assess the reliability of the questionnaire. To assess internal consistency, the Cronbach’s alpha was determined for a sample of 20 mothers, and to examine the test-retest stability, Intraclass Correlation Coefficient (ICC) was calculated for the same group of mothers, who had completed the questionnaire twice, with a two-week interval. Alpha coefficients higher than or equal to 0.06 were considered acceptable. ICC ≤ 0.4 were considered poor to fair agreement; 0.41–0.60 moderate agreement, 0.61–0.80 good agreement and 0.80 excellent agreement [19].

## Results

### Participants’ characteristics

A total of 265 mothers entered the study between June 10 and September 1, 2019. Participants’ mean (standard deviation) age was 27.66 (0.41) years with range of 16 to 43. The majority of them were housewife (95.1%), had a high school diploma (43.8%) and were primiparous (40.4%). Table 1 presents the other details of the participants.

**Table 1:** Characteristics of the study participants (*N* = 265)

Characteristics	N (%)
Age (years)^a^	27.66 (0.41)
College	44 (16.6)
Job	
Housewife	252 (95.1)
Employee	13 (4.9)
Income	
Not at all sufficient	34 (12.8)
Relatively sufficient	209 (78.9)
Completely sufficient	22 (8.3)
Unwanted pregnancy	100 (37.7)
Gestational age (weeks)^a^	37.90 (0.24)

^a^Mean (Standard Deviation)

### Content validity

In the face validity assessment, all the items in the questionnaire were described as simple and clear and achieved a minimum score of 1.5. In the content validity assessment, all the items achieved the acceptable values of CVI and CVR (Table 2).

**Table 2:** The impact score, CVI, and CVR for each questions of Respectful Maternity Care (RMC) (*n* = 265)

RMC	Impact score	CVI	CVR
RMC 1	4	1	1
RMC 2	4	1	0.8
RMC 3	3.90	1	1
RMC 4	4	1	1
RMC 5	3.90	1	0.8
RMC 6	3.93	1	1
RMC 7	3.93	1	1
RMC 8	3.93	1	1
RMC 9	4	1	1
RMC 10	3.96	1	1
RMC 11	3.90	0.96	1
RMC 12	3.93	1	1
RMC 13	3.96	0.96	0.8
RMC 14	3.93	1	1
RMC 15	3.90	1	0.8

*CVI* Content Validity Index, *CVR* Content Validity Ratio

### Construct validity

EFA was carried out on 15 items using the PCA. The KMO index was 0.945, and Bartlett’s test was 4129.019 at the significance level of *P* < 0.001, which justified the factor analysis according to the correlation matrix obtained from the study samples.

**Fig. 1:**
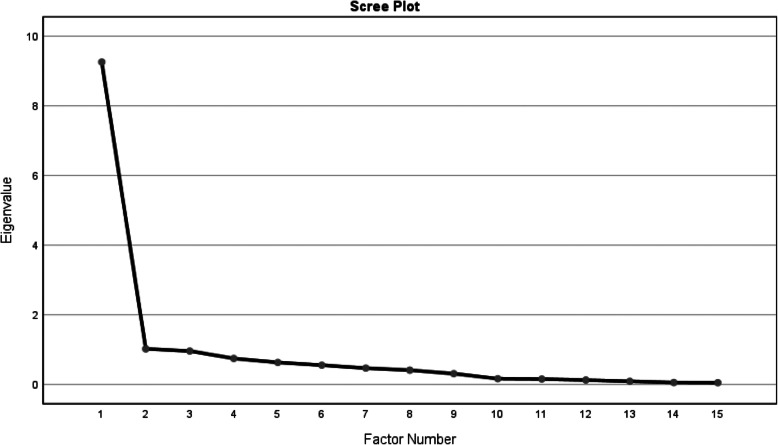
Scree Plot (Exploratory factor analysis for four factors of the questionnaire)

EVs and scree plots were used to determine the number of factors. The results obtained showed that the RMC questionnaire can be predicted by one factor considering that the highest percentage of total variance was 61.7%. Furthermore, based on the scree plot, one factor was set in the first descending slope of the plot (Fig. 1). This method thus also confirmed the model’s single-factor nature. Item 12 had a factor loading < 0.3 and was eliminated from this study. Item 12 would also be eliminated in the Principal Axis Factoring considering communalities < 0.2. A significant correlation was found between items 11 and 13 (*P* < 0.001), and these two items were thus integrated into one. Finally, the Persian version of the RMC questionnaire was approved with 13 items and one factor (Table 3).

**Table 3:** Factor loadings of the Respectful Maternity Care (RMC) (*n* = 265)

Items	Factor
RMC 1	0.946
RMC 2	0.956
RMC 3	0.899
RMC 4	0.928
RMC 5	0.953
RMC 6	0.602
RMC 7	0.498
RMC 8	0.683
RMC 9	0.674
RMC10	0.801
RMC 11	0.737
RMC 12	0.159
RMC 13	0.724
RMC 14	0.914
RMC 15	0.692

Extraction Method: Principal Axis Factoring

According to the indices presented in Table 4, the following values were obtained: X^2^/df < 5, RMSEA =0.08 and RMR < 0.1, which confirm the validity of this model. Furthermore, fit indices including GFI and AGFI were > 0.08, and TLI, NFI, RFI, IFI and CFI were > 0.9. This model had a favorable fit and its factor structure can thus be confirmed. Considering that the confirmatory factor model had a good relative fit and since the results showed a significant relationship between the tool’s items, the results of the exploratory factor model were supported by confirmatory models and the construct validity of the tool was thus confirmed.

**Table 4:** Confirmatory factor analyses fit Index of the Respectful Maternity Care (RMC) (*n* = 265)

Fit Indices (RMC)	Fit
$$ \raisebox{1ex}{${x}^2$}\!\left/ \!\raisebox{-1ex}{$ df$}\right. $$	2.94
RMSEA	0.08
GFI	0.88
AGFI	0.84
NFI	0.93
RFI	0.92
IFI	0.95
TLI	0.95
CFI	0.95

### Reliability

The Cronbach’s alpha coefficient was found as 0.93, which suggests the favorable internal consistency of the questionnaire. ICC (95% confidence interval) was determined as 0.98 (096 to 0.99) (Table 5).

**Table 5:** Mean (SD), Cronbach’s alpha, and ICC for the Iranian version of the Respectful Maternity Care (RMC)

Scales	Mean (SD)	Possible range	Obtainable range	Cronbach’s alpha	ICC (95% CI)^**a**^
RMC	61.31 (12.57)	20–74	15–75	0.93	0.98 (0.96 to 0.99)

^a^*ICC (95% CI)* Intraclass Correlation Coefficient (95% Confidence Interval)

## Discussion

According to the literature review, the psychometric properties of the Persian version of the RMC questionnaire have never been assessed. The present study was therefore conducted to assess the psychometric properties of this tool in a sample of Iranian mothers. The result of the study indicated that the RMC scale was a valid and reliable instrument to assess RMC in Iran. The validity and reliability of the questionnaire were confirmed.

The EFA of the RMC questionnaire showed that item 12 had a low factor loading, and it was therefore eliminated from the Persian version. The psychometric assessment of the Persian version led to the extraction of only one factor, while Sheferaw et al. (2016), extracted four factors (friendly care, abuse-free care, timely care and discrimination-free care) in their assessment of the psychometric properties of the original RMC questionnaire. In Sheferaw’s study, these four components showed a low correlation coefficient, which was considered a strong evidence for the tool’s construct validity [11]. The Cronbach alpha coefficient for the psychometrically-assessed questionnaire constructs was 0.93 in Iran, and like the psychometrically-assessed questionnaire of Sheferaw’s study, this figure suggests a favorable internal consistency.

In most studies, RMC has been assessed by interviews and qualitatively or by observation, and there is little quantitative information available on this tool. Plus, the little quantitative information available on it is dichotomous, i.e. with ‘Yes’ or ‘No’ responses [20–23], while the questionnaire designed by Sheferaw is quantitative, with responses based on a five-point Likert scale (from ‘totally agree’ to ‘totally disagree’). Another dichotomous quantitative questionnaire with ‘Yes’ or ‘No’ responses has also been designed by Abuya et al. (2015) to assess the prevalence of D&A in maternity facilities in Kenya. This tool was developed after a review of literature with a focus on four normative building blocks: (1) Human rights law: Physical abuse, non-confidential care; (2) Domestic law: Corruption and bribery; (3) Ethical codes: Non-consented care, abandonment and detention in facilities; and (4) Local consensus on behaviors: Non-dignified care [20]. Another tool, mother on Respect index (MOR), has been designed by Vedam et al. (2017) focused on mother-provider interaction and women’s sense of comfort and experiences of discrimination [24]. In Iran, there are two questionnaires that asses woman’s experience of RMC [25, 26]. The first relevant questionnaire was developed by Taavoni et al. (2018) based on seven categories of WHO on RMC. They have developed an instrument as the 59-items for evaluating RMC [25]. The second questionnaire was developed by Ayoubi et al., (2020) with a review of scientific literature and focus group discussion. Ultimately, their questionnaire has 19 items in three factors including providing comfort, participatory care and mistreatment [26].

In Sheferaw et al. study, four factors (friendly care, abuse-free care, timely care and discrimination-free care) were extracted. “Friendly care” is the first factor. This factor that had the largest number of items includes kindly and positively approach. This factor is similar to item of woman’s perception RMC (WP-RMC) tool developed by Ayoubi et al. including “providing comfort” [26]. The second factor is “abuse free care”. This factor includes physical and verbal abuse. This factor includes items mentioned in White Ribbon Alliances (WAR) rights [27] and Abuya et al. [20]. The third factor is “timely care”. It mentioned in delaying and waiting for receiving services. This factor is close to “providing comfort” in (WP-RMC) of Ayoubi et al. [26]. The last factor is “discrimination free care” that it point to personal attributes. This factor has certain commonalities with Vedam’s questionnaire [24].

Nonetheless, according to Sheferaw, other RMC parameters, such as consensual care, confidential care and the absence of abandonment and detention, have not been included in the questionnaire dimensions. The RMC questionnaire is therefore recommended to be combined with other questionnaires to enable the assessment of respectful maternity care in medical centers. By combining three tools, a comprehensive assessment of mother’s perception of RMC in Iran can be achieved.

### Strengths and limitations

The inclusion of multiparous and primiparous mothers with both term and preterm or singleton and twin pregnancies who had given birth by vaginal delivery is considered a strength of this study, because the psychometric assessment of the tool enables its use for all these groups. Another strength is concurrent sampling of both public and private hospitals. One of the limitation of this study was the selection of women from a single city (Tabriz). This limitation may decrease the generalizability of the findings in this study. The validity and reliability of the questionnaire needs to be re-assessed in other parts of Iran including rural areas to capture people input with diverse cultures. Another limitation of this study was that the participants who entered the study for confirmatory construct validity were the same people who complete the questionnaire for exploratory validity. To enhance the external validity of the study, researchers can carry out analyses on other datasets; however, a large sample must be selected and divided into two sub-samples to estimate stable parameters. The other limitation is that the questionnaire was completed by participants during 6–18 h after childbirth; whereas the data in the original questionnaire developed by sheferaw (2010), were collected immediately after childbirth [11]. We still believe that 6–18 h may not impact the possibility of recall bias. Data collection in hospital setting may increase under-reporting of D&A due to fear of receiving inappropriate medical service. We reduced this limitation with reassuring the mothers about the anonymous data collection method within a private room. It is recommended that other researchers evaluate this scale in different times after childbirth.

## Conclusion

The results confirmed the validity and reliability of the Persian version of the RMC questionnaire for the assessment of postpartum maternity care. This tool enables the managers of healthcare centers to assess the mothers’ satisfaction and implement the necessary modifications for meeting their needs and reducing disrespectful behaviors toward them.

## Supplementary information

**Additional file 1 Appendix 1**. The Persian version of respectful maternity care scale.

**Additional file 2 Appendix 2**. The English version of respectful maternity care scale.

## Data Availability

Not applicable.

## Abbreviations

D&A: Disrespect and Abuse
RMC: Respectful Maternity Care
WHO: World Health Organization
CVI: Content Validity Index
CVR: Content Validity Ratio
ICC: Intra Correlation Coefficient
EFA: Exploratory Factor Analysis
CFA: Confirmatory Factor Analysis
KMO: Kaiser-Meyer-Olkin
RMSEA: Root Mean Square Error of Approximation
