# Supplementary material for: The psychometric properties of the respectful maternity care (RMC) for an Iranian population
Source: BMC Health Serv Res. 2020 Sep 22;20:894. doi: 10.1186/s12913-020-05729-x (PMC7510070; doi:10.1186/s12913-020-05729-x)
Supplement: Supplementary file 1 — Additional file 1 Appendix 1. The Persian version of respectful maternity care scale. [file 12913_2020_5729_MOESM1_ESM.docx]

**پرسشنامه مربوط به مراقبت مادری احترام­آمیز**

1- کارکنان سلامتی از من به صورت محبت­آمیز مراقبت نمودند.

کاملاً موافقم 🞏 موافقم🞏 نمی­دانم و یا تفاوتی ندارد 🞏 مخالفم🞏 کاملاً مخالفم 🞏

2- کارکنان سلامتی با من رفتاری دوستانه داشتند.

کاملاً موافقم 🞏 موافقم🞏 نمی­دانم و یا تفاوتی ندارد 🞏 مخالفم🞏 کاملاً مخالفم 🞏

3- کارکنان سلامتی به صورت مثبت در مورد درد و تسکین آن صحبت نمودند.

کاملاً موافقم 🞏 موافقم🞏 نمی­دانم و یا تفاوتی ندارد 🞏 مخالفم🞏 کاملاً مخالفم 🞏

4- کارکنان سلامتی نسبت به من توجه و همدلی نشان دادند.

کاملاً موافقم 🞏 موافقم🞏 نمی­دانم و یا تفاوتی ندارد 🞏 مخالفم🞏 کاملاً مخالفم 🞏

5- رفتار تمامی کارکنان سلامت نسبت به من محترمانه بود.

کاملاً موافقم 🞏 موافقم🞏 نمی­دانم و یا تفاوتی ندارد 🞏 مخالفم🞏 کاملاً مخالفم 🞏

6- کارکنان سلامت با من با زبانی صحبت نمودند که من میتوانستم آن را درک نمایم.

کاملاً موافقم 🞏 موافقم🞏 نمی­دانم و یا تفاوتی ندارد 🞏 مخالفم🞏 کاملاً مخالفم 🞏

7- کارکنان سلامت مرا با اسمم خطاب­کردند.

کاملاً موافقم 🞏 موافقم🞏 نمی­دانم و یا تفاوتی ندارد 🞏 مخالفم🞏 کاملاً مخالفم 🞏

8-کارکنان سلامت به نیازهای من (درخواست شده یا نشده من) پاسخ دادند.

کاملاً موافقم 🞏 موافقم🞏 نمی­دانم و یا تفاوتی ندارد 🞏 مخالفم🞏 کاملاً مخالفم 🞏

9-کارکنان سلامت به دلایل مختلف در طول زایمان به من سیلی زدند.

کاملاً موافقم 🞏 موافقم🞏 نمی­دانم و یا تفاوتی ندارد 🞏 مخالفم🞏 کاملاً مخالفم 🞏

10-کارکنان سلامت به دلیل اینکه مطابق گفته های انها عمل نکرده بودم، سرم داد کشیدند.

کاملاً موافقم 🞏 موافقم🞏 نمی­دانم و یا تفاوتی ندارد 🞏 مخالفم🞏 کاملاً مخالفم 🞏

11-برای دریافت خدمات، مدت طولانی (بدون دلیل) منتظر نگه داشته شدم.

کاملاً موافقم 🞏 موافقم🞏 نمی­دانم و یا تفاوتی ندارد 🞏 مخالفم🞏 کاملاً مخالفم 🞏

12-در مرکز به من اجازه داده شد تا آیین های فرهنگی و مذهبی­ام (آوردن کتاب قرآن، بستن مچ بندهای مذهبی و ....) را انجام دهم.

کاملاً موافقم 🞏 موافقم🞏 نمی­دانم و یا تفاوتی ندارد 🞏 مخالفم🞏 کاملاً مخالفم 🞏

13-به دلیل مشکلات داخلی موجود در مرکز (کمبود کادر، کمبود تجهیزات و ....) ارائه خدمات به تاخیر می افتاد.

کاملاً موافقم 🞏 موافقم🞏 نمی­دانم و یا تفاوتی ندارد 🞏 مخالفم🞏 کاملاً مخالفم 🞏

14-کارکنان سلامت به دلیل ویژگیهای شخصیتی­ام (روستایی بودن و ....) با من به خوبی رفتار نکردند.

کاملاً موافقم 🞏 موافقم🞏 نمی­دانم و یا تفاوتی ندارد 🞏 مخالفم🞏 کاملاً مخالفم 🞏

15-کارکنان سلامت به دلیل ویژگیهای شخصیتی­ام (روستایی بودن و ....) به من و همراهانم توهین کردند.
کاملاً موافقم 🞏 موافقم🞏 نمی­دانم و یا تفاوتی ندارد 🞏 مخالفم🞏 کاملاً مخالفم 🞏
