# Supplementary material for: The psychometric properties of the respectful maternity care (RMC) for an Iranian population
Source: BMC Health Serv Res. 2020 Sep 22;20:894. doi: 10.1186/s12913-020-05729-x (PMC7510070; doi:10.1186/s12913-020-05729-x)
Supplement: Supplementary file 2 — Additional file 2 Appendix 2. The English version of respectful maternity care scale. [file 12913_2020_5729_MOESM2_ESM.docx]

**Respectful maternity care scale**

The following questions are used to assess your experience during labor and delivery. Please provide your agreement or disagreement to each question on a scale of 1 to 5. If you agree strongly to the statement use 5, agree to a statement use 4, if you are not sure or don’t know use 3, if you don’t agree to a statement use 2, and if you strongly don’t agree use 1.

Friendly care

Q1: The health worker/s cared for me with a kind approach

Q2: The health worker/s treated me in a friendly manner

Q3: The health worker/s talked positively about pain and relief

Q4: The health worker/s showed his/her concern and empathy

Q5: All health worker/s treated me with respect as an individual

Q6: The health worker/s spoke to me in a language that I could understand

Q7: The health worker/s called me by my name

Abuse free care

Q8: The health worker/s responded to my needs whether or not I asked

Q9: The health worker/s slapped me during delivery for different reasons (R)

Q10: The health worker/s shouted at me because I hadn’t done what I was told to do (R)

Timely care

Q11: I was kept waiting for a long time before receiving service (R)

Q12: I was allowed to practice cultural rituals in the facility

Q13: Service provision was delayed due to the health facility’s internal problems (R)

Discrimination Free care

Q14: Some of the health workers did not treated me well because of my personal attributes (R)

Q15: Some health workers insulted me and my companions due to my personal attributes (R)

(R): The item is reverse coded

Each dimension is calculated as follows.

1. Friendly care: sum (Item 1 to Item 7) X 100/35

2. Abuse free care: (Item 8 +item 9+Item 10) X 100/15

3. Timely care: (item 11+item12+ item13) X 100/15

4. Discrimination free care: (item14+item 15)*100/10
